# Genome sequence of *Cetobacterium somerae* ATCC BAA-474 isolated from human feces

**DOI:** 10.1128/mra.01196-24

**Published:** 2025-05-28

**Authors:** Saisuki Putumbaka, Claire E. Barrow, Farris L. Poole, Michael W. W. Adams

**Affiliations:** 1 Department of Biochemistry & Molecular Biology, University of Georgia 1355, Athens, Georgia, USA; University of Maryland School of Medicine, Baltimore, Maryland, USA

**Keywords:** *Cetobacterium somerae*

## Abstract

*Cetobacterium somerae* ATCC BAA-474 was first isolated from human feces and is emerging as an important species for animal and human health. Here, we report the complete genome sequence of this strain of *C. somerae* to improve the currently available genome sequence.

## ANNOUNCEMENT

*Cetobacterium somerae* strain ATCC BAA-474 was first isolated anaerobically at 37 °C using minimal salts-biotin media from human feces (1–4). It currently has a 3.068 Mbp draft genome assembly available that encodes approximately 2,800 genes in 174 scaffolds (ASM47904v1). This poor assembly makes genome-wide analyses and future genetic modifications difficult. *C. somerae* is prevalent in 68% of healthy human gut samples (5), and the increased abundance of this strain is a non-invasive biomarker for colorectal cancer (2). To better understand the complete metabolic capacity and the role *C. somerae* may play in the human gut microbiome, we re-sequenced and circularized the genome.

*C. somerae* strain ATCC BAA-474 was obtained from the DSMZ. Genomic DNA was extracted using the Quick-DNA Miniprep Kit (Zymo Research) from a clonal culture grown in anaerobic media supplemented with 5 g/L yeast extract (6) for 4 hours at 35 °C. gDNA (>50 ng/µL) quality was checked for non-fragmented high molecular weight DNA. The library preparation and sequencing were performed by Plasmidsaurus (Eugene, OR, USA). Default parameters were used for all software unless otherwise specified. Sequencing was performed using both Oxford Nanopore Technologies (ONT) long read and Illumina short read for optimal whole genome sequencing. For ONT long-read sequencing, library preparation was done using Rapid Barcoding Kit 96 V14, SQK-RBK114.96, and sequencing was performed on a PromethION P24 machine with an R10.4.1 flow cell equipped with base caller Dorado version 7.1.4 in super-accurate mode. Read quality control parameters were set to a minimum Qscore of 10, and adapters were trimmed by MinKNOW. For Illumina short-read sequencing, library preparation was done using the ExpressPlex 96 Library Prep Kit (SeqWell) and was sequenced on a NextSeq2000 machine using paired-end 2 × 150 bp settings. The total number of raw reads obtained was 174,739, and the mean read length was 3,639. The lowest quality 5% FASTQ reads were filtered out for both the short-read library and long-read library using Filtlong version 0.2.1 (7). The genome was assembled, circularized, and polished using Flye version 2.9.4 (8) with parameters for high-quality ONT reads.

The completeness of the assembly was assessed using CheckM version 1.2.2 (9) and shown to be 98% complete with 0.0% contamination. Genome quality was assessed using Quast (10), and the assembled *C. somerae* ATCC BAA-474 genome was shown to have an *N*_50_ of 1,938,920 and an *L*_50_ of 1. In comparison, the published NCBI genome (ASM47904v1) has an *N*_50_ of 47,157 and an *L*_50_ of 19. Genome annotation was performed using the NCBI Prokaryotic Genome Annotation Pipeline version 6.8 (11). The assembly revealed eight contigs (Table 1). The genome is a total of 3.2 Mbp and has 29% GC content. There are a total of 3,061 genes encoded within the genome. A summary of the new genome assembly is provided in Table 1. Contig 1 is the main chromosome. Using geNomad version 1.8.1 (12), we found that contigs 4, 6, 7, and 8 had their own replication machinery, resembling plasmids.

**TABLE 1:** *C. somerae* ATCC BAA-474 assembly summary

Contig	Length (bp)	Coverage (×)	Orientation	GC%	GenBank accession no.
1	1,938,920	176	Circular	29.5	CP173065.2
2	687,705	117	Circular	28.6	CP173062.2
3	198,846	75	Circular	27.5	CP173060.2
4	162,863	57	Circular	27.9	CP173070.2
5	145,122	69	Circular	26.6	CP173063.2
6	28,508	134	Linear	27.6	CP173066.2
7	18,453	83	Linear	25.7	CP173069.2
8	2,443	217	Linear	27.1	CP173064.2

## Data Availability

The genome assembly (GCA_044784365.2) was deposited to NCBI, and the raw data were deposited to SRA under the BioProject ID PRJNA1180557 and BioSample accession SAMN44522929. The sequences and annotations are deposited under GenBank CP173060.2-CP173070.2. Raw long-read data and short-read data are available in the SRA (SRR31184670 and SRR33139345).
